# Changing paradigms in lower extremity reconstruction in war-related injuries

**DOI:** 10.1186/s40779-016-0080-7

**Published:** 2016-03-31

**Authors:** Margaret Connolly, Zuhaib R. Ibrahim, Owen N. Johnson

**Affiliations:** Department of Surgery, University of Maryland Medical Center, 22 S Greene St., Baltimore, MD 21201 USA; Department of Plastic and Reconstructive Surgery, Johns Hopkins University School of Medicine, 601 N Caroline St., Baltimore, MD 21287 USA; Johns Hopkins University School of Medicine, 601 N Caroline St., Baltimore, MD 21287 USA

**Keywords:** Limb salvage, Free flap, Pedicled flap, Lower extremity, Blast injury, Combat, Battlefield, Reconstruction

## Abstract

**Background:**

Ballistic high-energy trauma has substantially increased the severity of non-fatal extremity injuries incurred in modern warfare. Expedient medical care, refinement in surgical techniques, and soft tissue coverage have brought about a paradigm shift in the management of lower extremity wounds during the last decade with an increased emphasis on limb salvage.

**Methods:**

A literature-based study was conducted to analyze reconstructive modalities based on the location, depth, and severity of wounds, as well as mechanism of injury, concomitant vascular injuries and open fractures, choice of flap, timing of definitive reconstruction, and complications.

**Results:**

Extremity injuries account for over 60 % of injuries in the recent conflicts in Iraq and Afghanistan, with the majority secondary to explosive devices. The severity of these injuries is profound compared with civilian registries, and conventional injury scoring systems have failed to accurately predict outcomes in combat trauma. The mainstay of treatment is serial debridement, negative pressure therapy, fracture stabilization, and treatment of concomitant injuries by the forward medical teams with subsequent definitive reconstruction after transport to an advanced military treatment facility. Autologous reconstruction with free tissue transfer and pedicled flaps remains the primary modality for soft tissue coverage in limb salvage. Adjunct innovative modalities, such as external tissue expansion, dermal substitutes, and regenerative matrices, have also been successfully utilized for limb salvage.

**Conclusion:**

Lower extremity injuries account for the vast majority of injuries in modern warzones. Explosive devices represent the most common mechanism of injury, with blast impact leading to extensive soft tissue injuries necessitating complex reconstructive strategies. Serial debridement, negative pressure therapy, and autologous reconstruction with free tissue transfer and pedicled flaps remain the mainstay of treatment in recent conflicts.

## Background

Lower extremity reconstruction following modern battlefield trauma poses significant challenges for the reconstructive surgeon compared with lower extremity injuries seen in the civilian population [1, 2]. High-energy blast impact from improvised explosive devices (IEDs) leads to an extensive zone of injury, which often results in limited donor site options for reconstruction [3, 4]. Inadequate perfusion, heavily contaminated open fractures, exposed bone with stripped periosteum, and devitalized tendons add to the complexity of these cases [5, 6]. Although amputation is inevitable, and even the preferred treatment in certain cases [7], successful limb salvage with optimal functional recovery can make a remarkable difference in the quality of life for this relatively young and otherwise healthy population. With technological advancements, limb salvage has been reported in as high as 93 % of patients from recent conflicts with high-energy ballistic injuries [1, 8].

Early return to ambulation, complete bone healing, and durable soft tissue coverage are key goals of successful limb salvage in complex lower extremity trauma [7, 9]. In this context, the concept of the reconstructive ladder exists as a guide for choosing the appropriate method of reconstruction in civilian injuries [10]. However, highly encouraging reconstructive results from innovative modalities, such as dermal substitutes, regenerative matrices, pedicled perforator flaps, and tissue expansion in the civilian population have made the decision making more complex and individualized [10, 11]. Despite abundant literature demonstrating successful soft tissue coverage using these advanced technologies in the civilian population, the utility of such modalities in the military setting has not been thoroughly examined. Additionally, contemporary reconstructive strategies may not be applicable to the extensive injuries from blast impacts in battlefield trauma.

## Review

### Methods

A literature-based study was conducted using PubMed, Cochrane, and Embase databases for articles published in English ranging from 2005 to 2015. This included the following search terms: “lower extremity,” “reconstruction,” “flap,” “military,” “combat,” and “battlefield.” For historical and technical background, articles published prior to 2005 were also reviewed if they described a historical perspective of currently utilized modalities. For the purpose of this review, articles limited to amputation or vascular repair without pertinent data on soft tissue reconstruction were excluded. Similarly, single patient case reports were excluded unless they described a significant adverse outcome related to a reconstruction modality.

### Results

The majority of contemporary scientific evidence for advanced treatment of combat-related lower extremity trauma comes from the current conflicts in Iraq and Afghanistan. The data sources for these studies include the Department of Defense Trauma Registry, US Navy and US Marine Corps Combat Trauma Registry Expeditionary Medical Encounter Database, and hospital-based data from military treatment facilities. In addition, a case-control study for lower extremity reconstruction of landmine-related injuries is included in this review [12]. Available literature for combat-related injuries consists of a retrospective cohort of patients with lower extremity wounds (Evidence Level 3).

#### Mechanism of injury

In contrast to civilian injuries, penetrating trauma accounts for the vast majority of lower extremity injuries, with 63.3 % of injuries resulting from explosive devices and 16.3 % from high-velocity gunshot wounds. These explosive devices also result in devastating blunt injuries in the form of blast effect, shockwave, intimal damage, and closed brain injury [13].

#### Timing of surgery

Godina [14] demonstrated that failure and complication rates of free-tissue transfer in civilian patients increase if the operation is performed more than 72 h after injury. However, there are significant differences in combat injuries, which can be contraindications to immediate free tissue transfer. In the senior author’s opinion, provision of definitive care in the battlefield poses several challenges and, hence, the US military doctrine is to stabilize and evacuate the wounded soldier with definitive reconstruction performed after transfer. In the recent conflicts, the average time from injury on the battlefield to arrival at a local tertiary medical center was 4 to 10 days [15]. Final disposition is reserved for when the patient is least critically ill and at the facility best-equipped for treatment and reconstruction. Combat wounds are associated with heavy contamination and devitalized tissue, usually requiring serial debridement, proper broad spectrum intravenous antibiotics, and complex fracture management prior to definitive coverage [2, 3]. Kumar et al. reported 12 free and 62 pedicled flaps performed in a sub-acute period after serial debridement with complete failure rates of 0 and 1.4 %, respectively [2]. In a later series, Kumar et al. reported 46 flaps (10 free and 36 pedicled) for lower extremity reconstruction with only one flap loss [3]. Hence, emergent revascularization, resuscitation, stabilization, fracture reduction, debridement, and negative pressure therapy with definitive flap coverage in a sub-acute period remains the mainstay of treatment.

Because traditional scoring systems such as the Mangled Extremity Severity Score (MESS) fail to accurately predict the outcome of limb salvage in ballistic wounds [7], the decision to perform an amputation versus limb salvage is reserved until evacuation and transport to an advanced treatment facility. Brown et al. [7] reported a retrospective series of 77 military personnel with 86 mangled ballistic lower extremity wounds and assessed the predictability of the MESS system. Traditionally in the civilian population, a MESS of 7 or greater is considered an indication for amputation [16]. However, this criterion resulted in positive predictive value of only 64.3 % in ballistic injuries, bringing its applicability in combat wounds into question [7]. According to this report, prolonged hypotension and ischemia were the strongest predictors of failed limb salvage in ballistic lower extremity injuries [7]. The MESS was also not helpful in predicting which patients referred to a limb salvage clinic would ultimately require late amputation [17].

Microvascular free tissue transfer for host nation patients was also performed within the combat support hospitals with over a 90 % success rate [4] in the sub-acute phase (within 1 month from the initial injury). In various parts of the world with limited resources and equipment, these results are more applicable for conflicts and add to the importance of microvascular training and expertise needed in military medicine, particularly at support hospitals.

#### Method of reconstruction

Autologous tissue transfer with either pedicled or free flaps has remained the pillar of treatment for definitive reconstruction in recent conflicts. Sabino et al. [18] reported 10 years of experiences at Walter Reed National Military Medical Center where 359 flaps were performed for war-related extremity trauma (143 free and 216 pedicled flaps), including poly-extremity trauma and reconstruction, with outcomes comparable to other military and civilian lower extremity reconstruction. In this series, 55 % of flaps were muscle flaps, and 42 % were fasciocutaneous flaps. The most common free muscle flap was the latissimus dorsi (13 %), the most common free fasciocutaneous flap was the anterolateral thigh (11 %), the most common pedicled muscle flap was the gastrocnemius (17 %), and the most common pedicled fasciocutaneous flap was the sural flap (6 %). Historically, a massive zone of injury in ballistic wounds was considered to be a precluding factor for a rotational flap; however, Burns et al. [19] demonstrated that pedicled flaps are a safe option for carefully selected cases of severely injured extremities. A case control study from the Turkish Armed Forces Rehabilitation Center demonstrated no significant differences in functional ambulation scale, energy expenditure, and a 10 min walking test when comparing free muscle flap coverage of severe landmine-related heel defects to healthy volunteers [12]. This highly encouraging functional outcome advocates for the use of autologous free tissue transfer for foot and ankle defects.

#### Concomitant vascular injuries and open fractures

Concomitant vascular injuries are common in ballistic lower extremity wounds. In a recent report, 24 % of patients requiring flap coverage for ballistic lower extremity wounds had concomitant vascular injury that required emergent repair [20]. Flap coverage was performed at an average of 31 days following vascular repair with an overall flap failure and complication rate of 8 and 31 %, respectively, which was comparable to the complication rate of flaps not requiring vascular repair (10 and 28 %, respectively). However, variability in the flap success rate exists among different series from a variety of centers and time periods, primarily based on the extent of lower extremity trauma, which became progressively worse in later time periods. Treatment strategies and technical skills also improved over time leading to heterogeneity in outcomes.

Dickens et al. [21] analyzed the risk factors for amputation versus limb salvage in open calcaneal fractures (102 open fractures: 43 amputations, 59 limbs salvaged) and reported a progressively increased risk of amputation with increased wound area. Unsuccessful limb salvage was also significantly associated with ipsilateral forefoot fracture, talar fracture, plantar wound, and positive wound culture. In this series, 15 % of patients had delayed amputation (greater than 12 weeks post-injury). In contrast, in the civilian population, the Lower Extremity Assessment Project (LEAP) study reported a delayed amputation rate of only 3.9 % [22]. Of note, lack of plantar sensation was not significantly associated with increased risk of amputation. Expert opinion on strategies for managing ballistic foot wounds also suggests a high likelihood of amputation in open calcaneal fractures with extensive plantar soft tissue loss [23, 24].

Similarly, Doucet et al. [13] compared open tibial fractures sustained in combat with a civilian registry and reported 21 amputations out of 115 (18 %) open tibial fractures in combat-related injuries compared with only 45 amputations out of 850 (5 %) civilian open tibial fractures. Although combat-related open tibial fractures are associated with a higher incidence of amputation, limb salvage was possible in almost 82 % of these injuries.

#### Adjunct reconstructive modalities

Innovative reconstructive strategies, such as dermal substitutes, regenerative matrices, and tissue expansion, are now commonly utilized in lower extremity reconstruction in civilian trauma [25]. Fleming et al. [26] demonstrated successful use of these modalities in single patient case reports, primarily used as an adjunct to traditional flaps for preserving limb length in war-related amputation stumps. In this context, a biologic composite dermal substitute (Integra Life Sciences Inc.) with staged skin was used to provide a pliable surface for prosthetic use while preserving a functional limb segment. In another retrospective series, 15 out of 16 wounds with significant soft tissue loss were successfully reconstructed with a bilayered dermal substitute and staged skin grafting [27]. Similarly, external tissue expanders were used for delayed primary closure to provide a pliable surface for prosthesis fitting [26].

Valerio et al. [11] reported a case series of 51 patients where a porcine urinary bladder matrix was applied as a biologic scaffold (MatriStem MicroMatrix®, ACell, Inc., MD, USA) to promote granulation over either tendon without paratenon or bone without periosteum. Success was seen in 86 % of wounds with MatriStem application that proceeded to full healing with skin graft, dermal substitute, or flap coverage. Although subjective observational findings demonstrated constructive remodeling and a healthy vascularized wound bed, the study had inherent limitations, including lack of a control group and varying numbers of procedures prior to application of the biologic scaffold.

Intraoperative indo-cyanine green laser angiography (ICGLA) has also been used as a guidance tool for complex debridement in heavily contaminated wounds, soft tissue avulsion injuries, amputation stumps, and flap design and assessment [28]. In a retrospective series, intra-operative plans were modified due to perfusion related issues in 35 out of 186 (18.8 %) patients, as detected by ICGLA. These reports provide promising results for future use of innovative modalities in war-related injuries.

#### Complications

Flap failure, hematoma, chronic pain, non-union, osteomyelitis, heterotopic ossification, and delayed amputation represent the major complications reported in war-related extremity reconstructions [5, 18]. Overall, the success rate for flap coverage exceeds 90 % [2–4, 18]. Sabino et al. [18] reported flap failure leading to amputation in only four out of 359 cases of flap coverage (1 %). There was no difference in the overall complication rate between muscle and fasciocutaneous flaps. Similarly, Huh et al. [5] reported 213 open (Gustilo-Anderson) [29] Type 3 tibial fractures out of which 177 were managed with limb salvage, while the remainder underwent primary amputation within 12 weeks of injury. Eleven out of 177 patients underwent late amputation with deep soft tissue infection and osteomyelitis as the most significant risk factors for late amputation. These patients with late amputation required a significantly higher number of revision procedures and had a higher incidence of flap failure secondary to deep infection. In a retrospective analysis of 964 US military personnel with major lower extremity injuries, Melcer et al. [30] found early amputees and limb salvage patients had comparable physical complication rates; however, limb salvage patients had significantly higher psychological diagnoses. Late amputees (>90 days) had significantly higher physical and psychological complications than both early amputees and limb salvage patients.

### Discussion

The mechanism of injury, depth, and location of the wound are important considerations when selecting a method of reconstruction for complex lower extremity wounds. However, regardless of the method of reconstruction, adequate debridement of devitalized tissue is critical for overall success of any reconstructive modality, and this is of particular importance for lower extremity wounds where tissue perfusion can be marginal [2]. Thorough debridement and irrigation is essential to removing foreign materials that may be embedded in the tissue secondary to blast injuries [31]. Inadequate debridement can lead to persistent infection, osteomyelitis, and delayed amputation. Such complications may persist despite aggressive and repeated debridement. Although innovative technologies, such as a bi-layered tissue regenerative matrix with staged skin grafts and tissue expanders, have been utilized in lower extremity reconstruction in civilian population [32], their utility as definitive coverage has yet to be determined in war-related injuries. Heavy contamination in combat zones and the presence of multi-level open fractures have precluded the widespread use of these technologies for commonly encountered ballistic injuries in recent conflicts.

War-related injuries are often associated with poly-extremity trauma. Multiple free flaps in the same patient for multiple limb salvage have demonstrated no significant difference in overall complication rate. However, the reported free flap failure rate risk in multiple limb salvage cases is significantly higher compared with single limb salvage [33].

With technological advances in resuscitative trauma care and fracture management, reconstructive options have evolved as well, with more injuries salvaged than ever before [7]. This further adds to the importance attempting limb salvage at the initial encounter. However, according to the Military Extremity Trauma Amputation/Limb Salvage (METALS) study [9], those treated with amputation had a better functional outcome than limb salvage at 3 years post-injury, with both groups demonstrating moderate to severe physical and psychosocial disability. Delayed physical and psychological complications may be related to differences in rehabilitation options for amputees and limb salvage patients. While limb salvage clinics exist, patients are typically referred only when the injured limb is at risk of late amputation [17]. In contrast, there are established military amputee care programs (ACPs) more readily available to amputees [30]. Differences in rehabilitation protocols, extent and severity of injury, and potential selection bias together present challenges in drawing a universal conclusion based on this study alone. In this context, elective amputation versus further reconstruction for limb salvage at definitive medical treatment centers plays a pivotal role after a thorough discussion of potential challenges, including prolonged recovery, persistent disability, and need for multiple revision procedures.

Pedicled perforator flaps based on perforasome theory are now commonly utilized for lower extremity reconstruction in the civilian population [34]. Due to extensive soft tissue injury and the potential violation of fascial planes and perforators in ballistic injuries, island flaps based on perforators are uncommon in war-related patients. Traditional pedicled flaps, such as the gastrocnemius muscle and sural fasciocutaneous flaps, however, are the most common methods of reconstruction in reported literature [3, 18].

The majority of current evidence is based on reports from US military medical centers where amputees undergo aggressive and state-of-the-art rehabilitation. These patients have access to advanced prosthetic devices, and amputation is a more socially acceptable option within this community [13, 35]. For the rest of the population, there may be logistic, social, and cultural reasons that lead to different functional and psychosocial outcomes following amputation compared with limb salvage. The mechanism and pattern of injuries may also differ based on evacuation systems, healthcare delivery systems, and the availability of advanced body armor. Global data on the outcome of combat-related lower extremity injuries in current conflicts outside of the studies discussed are limited, and the technical considerations on the choice of limb salvage versus amputation, as well as the choice and timing of flap, may depend on available resources and expertise.

## Conclusions

Advancements in body armor, multidisciplinary trauma resuscitation, and surgical care have resulted in improved combat casualty survival rates; however, they have also lead to an increase in non-fatal extremity injuries from explosive devices. Ballistic impact from these injuries often causes an extensive zone of injury with multi-level open fractures and soft tissue defects. Resuscitative care, serial debridement, negative pressure wound therapy, and fracture stabilization remain the mainstay of early treatment, while definitive coverage is performed at military treatment facilities in a sub-acute manner. Reconstructive modalities, such as dermal substitutes, regenerative matrices, and external tissue expansion, have been successfully explored as adjunct modalities in the treatment of war-related injuries. Pedicled and free tissue transfer, however, are consistently the primary methods of definitive soft tissue coverage. Both limb salvage and amputation are associated with moderate to severe functional disability. Short and intermediate term results have shown significantly improved functional results with early amputation when compared with limb salvage in combat trauma. However, the global impact of these results is unknown due to cultural, social, and logistic differences in aggressive rehabilitation and the availability of advanced prosthetic devices.

## Abbreviations

ICGLA: Indo-cyanine green laser angiography
IED: Improvised explosive device
LEAP: Lower Extremity Assessment Project
MESS: Mangled Extremity Severity Score
METALS: Military Extremity Trauma Amputation/Limb Salvage
